# Jejunal Intussusception: A Rare Presentation of Carcinoid Tumor

**DOI:** 10.1155/2015/260697

**Published:** 2015-06-29

**Authors:** Umashankkar Kannan, Amir A. Rahnemai-Azar, Ashish N. Patel, Vinaya Gaduputi, Ajay K. Shah

**Affiliations:** ^1^Department of Surgery, Bronx Lebanon Hospital Center, Albert Einstein College of Medicine, Bronx, NY 10457, USA; ^2^Department of Medicine, Bronx Lebanon Hospital Center, Albert Einstein College of Medicine, Bronx, NY 10457, USA

## Abstract

A 55-year-old male presented to the emergency department with sudden onset of diffuse abdominal pain for one day. Physical examination was remarkable for tenderness in the umbilical region. A CT scan of the abdomen showed intussusception involving the jejunum without any mass. The patient then underwent an exploratory laparotomy. During surgery, the distal jejunum was intussuscepted with mesenteric lymphadenopathy. Liver showed nodular deposits in both lobes of the liver. The involved small bowel segment was resected with primary anastomosis and liver was biopsied. Pathological examination showed multifocal deposits of well-differentiated carcinoids in the jejunum. The liver and mesenteric deposits were positive for metastatic carcinoid. Patient recovered well without any complications.

## 1. Introduction

Intussusception in adults is a rare occurrence accounting for 5–10% of all patients with intussusception and 1% of adults with bowel obstruction [1, 2]. Intussusception is the telescoping of a proximal segment of the gastrointestinal tract into an adjacent distal segment. Unlike children, adult intussusception has structural etiology in the majority of cases. Owing to the presence of nonspecific symptoms, it presents a unique challenge in management. We hereby present a case of jejunal intussusception in an adult secondary to carcinoid tumor presenting as small bowel obstruction.

## 2. Case Presentation

A 55-year-old male without any significant past medical history presented to the emergency room with 1-day history of sudden onset umbilical pain. He denied any nausea, vomiting, abdominal distention, or recent change in bowel habits. He had an unremarkable screening colonoscopy about 5 years ago. Physical examination was remarkable for soft, nondistended abdomen with tenderness over the umbilical region. Laboratory tests revealed hemoglobin 15.1 mg/dL, WBC 6800 mm^−3^, and platelet count 169,000 mm^−3^. Serum lactate at presentation was 2.9 mmol/L. The rest of the blood chemistry was within normal limits.

**Figure 1:**
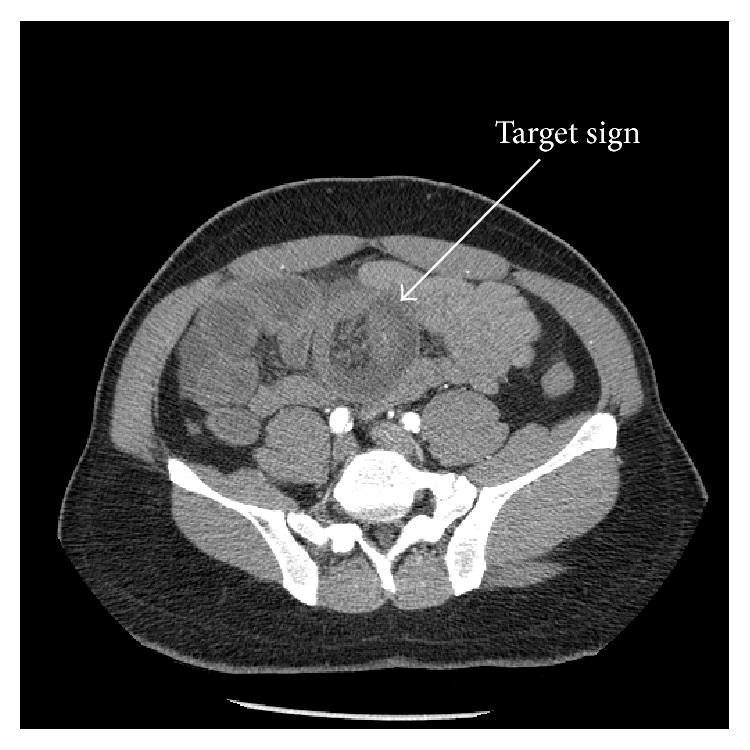
Tomographic view of the abdomen showing small bowel intussusception with the characteristic “target sign.”

**Figure 2:**
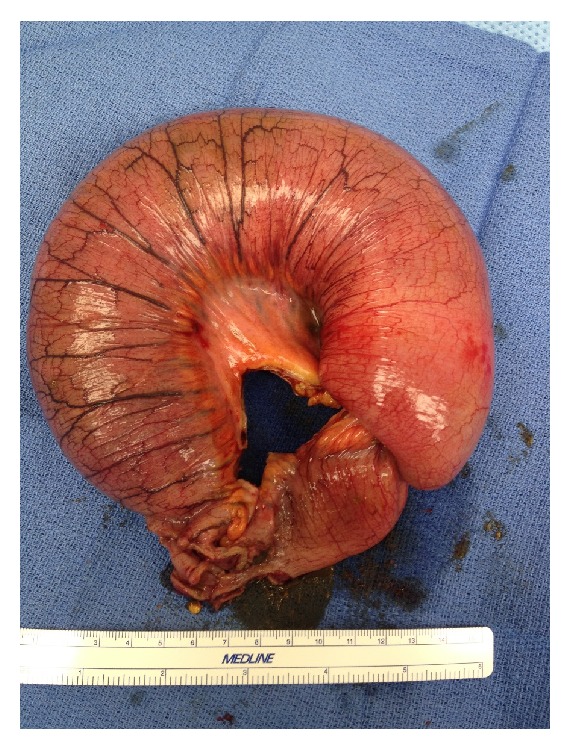
Operative specimen of small bowel intussusception.

Computed tomography (CT) of the abdomen suggested intussusception involving the jejunum without any mass as in Figure 1. Based on these findings, patient underwent exploratory laparotomy with findings of intussusception involving the jejunal segment with mesenteric lymphadenopathy (Figure 2). Liver had multiple nodular deposits involving both lobes. The small bowel segment involving the intussusception with 5 cm margin of adjacent normal intestine was resected and gastrointestinal tract was reconstituted. Postoperative period was uneventful.

Pathological examination of the surgical specimen showed 2 tumor nodules in the jejunum extending to the muscle layer consistent with well-differentiated neuroendocrine tumor. The largest nodule measured 2.5 cm and Ki-67 index was less than 2%. Mesenteric lymph node and liver biopsy were positive for metastatic neuroendocrine tumor. Immunological staining was positive for Chromogranin A and synaptophysin. Postoperatively 24-hour urine samples for 5-hydroxyindole acetic acid measured were elevated (10.8 mg/L). In 6-month postoperative follow-up visit, patient is doing well without any complications.

## 3. Discussion

About 80–90% of the intussusceptions in adults have identifiable etiology and the most common site is the small bowel [3]. Malignant etiology is identified in about 25% of the small bowel intussusceptions (SBI) and 48% of the colonic intussusceptions (CI) [4]. The most common malignant lead point in SBI is the metastasis, with melanoma being the most common. Other malignant causes include lymphoma, metastasis from colon, lung, and kidney, primary adenocarcinoma, and other less common malignancies [4]. In our patient, the lead point was the multifocal carcinoid tumor of the jejunum.

Carcinoid tumor is the most common small bowel tumor accounting for 20–50% of all small bowel tumors. They arise from the enterochromaffin cells (Kulchitsky cells). They are characterized by excessive production of peptides, neuramines, and other vasoactive substances. Carcinoid syndrome resulting from excessive secretion of these substances presents as flushing, diarrhea, right sided valvular disease, and bronchial constriction. Over 20 years between 1985 and 2005, the proportion of reported small bowel carcinoids has increased from 27.5% to 44.3% [5]. Carcinoid tumors are more frequently (44% of cases) identified in the ileum, multifocal in 25% of cases and distant metastases in 16% of cases at presentation [5, 6].

The most common symptom is the abdominal pain which is often crampy and paroxysmal. Bowel obstruction is another common manifestation. In an analysis of small bowel tumors in emergency surgery [7], 20% were secondary to carcinoids and all of the cases presented as bowel obstruction. Carcinoids can also manifest like massive lower intestinal bleeding and like mesenteric ischemia. The index case presented as small bowel obstruction secondary to jejunal intussusception. In our review of the English literature there was just one report of jejunal intussusception [8]. Ileal carcinoids presenting as intussusception have been reported more frequently [9]. Liver is the most common site of small bowel carcinoids as seen in our patient.

The World Health Organization has recently released a binary system classifying the neuroendocrine tumors to well-differentiated and poorly differentiated neoplasms [10]. About 99% of the small bowel carcinoids are well differentiated as in our case.

In adults, CT is the main diagnostic modality that shows the pathognomonic “target” sign. In addition, it excludes other competing differential diagnoses. In a single institutional study, CT has been shown to have a sensitivity of 86%, a specificity of 100%, and an accuracy of 89% for the diagnosis of carcinoid tumor. Other modalities include magnetic resonance imaging (MRI) and somatostatin-receptor scintigraphy (octreotide scan). Carcinoid tumors produce functional hormones and inactive proteins such as Chromogranins. Measurement of 24-hour urinary 5-hydroxyindole acetic acid (5-HIAA) is usually normal in localized tumors and elevated in liver metastasis. Serum Chromogranin A level is elevated in well-differentiated carcinoid tumors and correlates with tumor burden [6].

In pediatric patients, reduction of intussusception alone without resection is sufficient. However, almost 90% of intussusception cases in adults are secondary to a pathologic condition and resection is often recommended treatment of choice due to significant risk of associated malignancy [3]. Reduction of the intussusception before resection is tempting and theoretically will limit the amount of the resection. However, except in certain cases (like leading point with benign nature), this should be avoided because of the risk of potential intraluminal seeding or venous tumor dissemination during manipulation of malignant lesion [11]. In the case of resection, removal of the involved segment with associated mesenteric nodes is essential to meet standard complete oncologic resection. In some cases like Peutz-Jeghers syndrome because of the recurrent nature of the intussusception initial reduction during surgery followed by enterotomy and polypectomy is recommended [3]. In case of liver metastasis, treatment ranges from resection or ablation to transarterial chemoembolization and liver transplantation [6]. Systemic therapy for advanced diseases includes treatment with somatostatin analogues like octreotide and lanreotide. Other therapies include treatment with mTOR inhibitors, vascular endothelial growth factor (VEGF) pathway inhibitors, and cytotoxic chemotherapy [6].

## 4. Conclusion

Intussusception in adults is rare and differs from pediatric population. Most of the small bowel intussusceptions have a structural etiology and a small proportion of them harbor malignancy. Small bowel carcinoids are group of slow growing well-differentiated tumors. Diagnosis can be made preoperatively with CT and USG being the common modalities. Surgery is the mainstay of treatment of small bowel carcinoids. Liver directed and systemic therapies are used in advanced diseases.
